# Alternative Use of Femoral Compression System in Palliative Care: A Case Report

**DOI:** 10.7759/cureus.59827

**Published:** 2024-05-07

**Authors:** Gabriela Alonso, Priscilla Tubbs, Mery Lossada

**Affiliations:** 1 Internal Medicine, Nova Southeastern University Dr. Kiran C. Patel College of Osteopathic Medicine, Clearwater, USA; 2 Internal Medicine, DeBusk College of Osteopathic Medicine, Lincoln Memorial University, Harrogate, USA; 3 Palliative Care, Hospice of Marion County, Ocala, USA

**Keywords:** gynecologic oncology, community-based palliative care, arterial bleed, terminal hemorrhage, symptom control, symptom management at the end of life

## Abstract

Terminal hemorrhage, although relatively rare, presents a significant challenge in palliative care, necessitating effective management strategies to alleviate distress in patients and their families. This report presents the case of a 77-year-old woman with advanced vulvar squamous cell carcinoma. The patient had ulcerated groin metastases that had affected the nerve and vasculature of her left leg. Upon clinical examination, she had a large nonhealing ulcer of 11 cm x 2 cm x 4 cm on her upper left thigh that contained slough and was oozing. After a goals of care discussion with the patient and her family, she was admitted to hospice for comfort care measures. This case highlights an unconventional use of a femoral compression device in the palliative care setting, offering a novel approach to managing terminal hemorrhage. Commonly, these devices are used for hemostasis following different procedures, such as vessel cannulation or femoral artery pseudoaneurysm repair.

## Introduction

With a reported incidence of 3%-12%, terminal hemorrhage, defined as major bleeding from an artery, is likely to result in death. Although the incidence is likely higher in head and neck cancers because carotid artery rupture is the most common complication, it is important to be mindful of the possibility of this complication in any major artery. Exsanguination that leads to terminal hemorrhage carries a psychological burden for patients, their families, and their healthcare providers [1]. The current guidelines to manage hemorrhage include using both local and systemic options. Local measures include adjusting the patient's posture, using compression dressings, packing the wound, applying hemostatic agents, using radiation therapy, and performing embolization. Systemic measures include transfusions of packed red blood cells, plasma, and platelets, administration of vitamin K, and use of antifibrinolytic agents [2]. We present a case where a FemoStop (Abbott, Chicago, IL), a femoral vessel compression device, was used to prevent potential exsanguination in a patient receiving end-of-life care in our inpatient hospice unit. These devices are designed for rapid recovery and minimization of complications in patients who have undergone a cardiac catheterization [3]. They vary in construction, but the device we used is usually left on for one to two hours to control bleeding after vessel cannulation or pseudoaneurysm repair. They have also been used in both emergency medicine and trauma settings to control bleeding [4,5]. Here, we describe an alternative use of this device in palliative care at the end of life.

## Case presentation

We presented the case of a 77-year-old Caucasian woman with terminal squamous cell carcinoma of the vulva. The patient had ulcerated groin metastases that had affected the nerve and vasculature of her left leg. Her pertinent past medical history included a provoked pulmonary embolism, lymphoma, and unspecified asthma. Her past surgical history included a vulvar carcinoma resection and excisional debridement of her left groin just a few weeks before.

**Figure 1:**
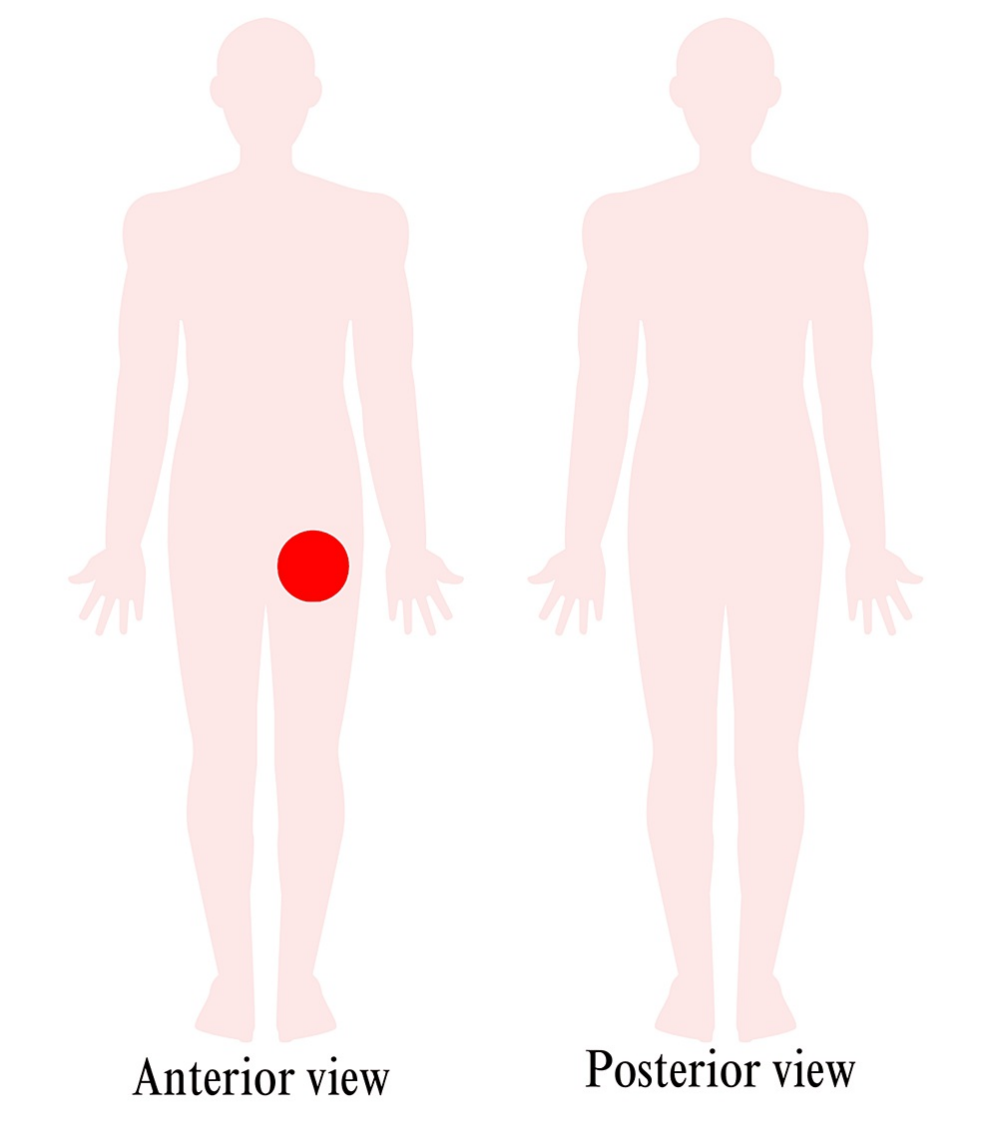
Physical examination findings Red circle: patient's site of ulceration

On March 29, 2023, she arrived at the emergency department from the hospice inpatient unit per her husband’s request due to a change in goals of care. She presented with worsening mentation, increased pain, and bleeding. Upon examination, her vitals were stable (Table 1), and she appeared uncomfortable secondary to pain. She had a large nonhealing ulcer on her upper left thigh, which measured 11 cm x 2 cm x 4 cm, contained slough, and was oozing (Figure 1). Vascular surgery was consulted. The patient was evaluated, and no wound care or further intervention was recommended due to the high risk of exsanguination. Radiation oncology was also consulted, where palliative radiation treatment was recommended. However, the patient's family decided not to proceed with that option.

**Table 1:** Initial vital signs in the emergency department

Vital signs	Results
Pulse Ox	98%
O_2_ delivery	Room air
Blood pressure	106/52
Heart rate	70
Respiratory rate	15

On March 30, 2023, after a discussion with the patient and her family, she was readmitted to hospice and transferred to the inpatient unit for pain management and close observation due to her high risk of exsanguination. Upon examination, the patient had decreased breath sounds bilaterally and was unresponsive. Her abdomen was globose, with normoactive bowel sounds. There were multiple dressings with bright blood on her left groin area. The dressings were removed and a pack of clean gauze was placed on the wound. The FemoStop, a femoral compression device, was then placed over the center of the wound, and the device was inflated to 30 mmHg. This was expected to maintain the patient’s comfort while preventing exsanguination. Comfort measures and pain management were continued for the next seven days until the patient expired.

## Discussion

The quality of life of patients with advanced cancer is significantly affected by a complex symptom burden. Although rare, terminal hemorrhage may be a complication of advanced cancer. Bleeding distresses the patients and their families.

This case report explores the role of a mechanical compression device in controlling femoral artery bleeding in the palliative care setting. This device is used in the compression of femoral arteries or veins after catheterization. It has also been used to control bleeding in both emergency medicine and trauma settings [4,5]. When used for its indication, the device is inflated to 10-20 mmHg above the systolic blood pressure and then lowered to a maintenance pressure after three minutes to maintain limb perfusion. In this off-label use, the device is inflated to 30 mmHg and is maintained at that level after peripheral pulses are checked. This is preferable to the recommendation to cease intervention or dressing changes because it protects her open wound, ultimately reducing the risk of bleeding. Another case report by Cambron et al. [4] demonstrated the use of a FemoStop in a patient with an erosion that extended into his femoral artery. The patient began to bleed, and attempts to control his bleeding with transfusions, tranexamic acid, DDAVP, and manual pressure were unsuccessful. Hemostasis was immediately achieved with the FemoStop. The application of the FemoStop on the patient's arterial bleed provided protection for the patient’s open wound and reassurance to the patient and her family that she would not exsanguinate.

## Conclusions

This article emphasizes that while there are not many trials available on managing bleeding in a patient in the palliative care setting, management should be based on personal preference, available resources, and cost. The general approach involves discussing with patients and their families, and using local measures (packing, compression dressings, and topical hemostatic agents) and systemic measures (antifibrinolytics, packed red blood cells, plasma, and vitamin K). Palliative radiation treatment is another option to manage bleeding. Currently, dark towels and linens are used in hospice inpatient units to help amend the stress of bleeding in a patient. Overall, we received positive feedback from healthcare providers and staff regarding the use of the FemoStop in managing the patient’s bleeding. The device is relatively easy to use and provides a level of comfort regarding the prevention of exsanguination to everyone involved in the patient’s care. A few months after this case, the hospice team had another patient with an arterial bleed and used the FemoStop again with similar positive results.

While this case report introduces another option available to providers, more research needs to be done to investigate the most effective way to manage arterial bleeding in palliative care patients. This may be difficult in the palliative care setting due to the challenges of conducting randomized controlled trials.
